# Implementation of formative assessment and its effectiveness in undergraduate medical education: an experience at a Caribbean Medical School

**DOI:** 10.15694/mep.2018.0000131.1

**Published:** 2018-06-13

**Authors:** Sateesh Babu Arja, Yogesh Acharya, Sabah Alezaireg, Vishnupriyan Ilavarasan, Samprith Ala, Sireesha Bala Arja

**Affiliations:** 1 Avalon University School of Medicine

**Keywords:** Medical Education Research, Self-assessment, Assessment, Feedback, Teaching and Learning

## Abstract

This article was migrated. The article was marked as recommended.

Background:

Two types of assessments used in the medical education are summative and formative assessments. Summative assessments evaluate a student’s learning progress and typically provide concrete grades or pass/fail decisions. The main purpose of the formative assessments is to provide the feedback and improve the learning behavior of the students. However, it is less known about the impact of the formative assessments on overall student’s performance in summative assessments. This study is intended to study the effects after the implementation of formal formative assessments on students overall performance in summative assessments or any changes in the total grade point average in a Caribbean Medical School which is a low resource context.

Materials and Methods:

A longitudinal cohort study was conducted among the basic sciences students at AUSOM in May 2017. The study was supported by quantitative data, quantitative and qualitative questionnaires to address the objectives of the study. Data collected and analyzed with Stata (Stata 15).

Results:

58, among 67 total students took part in the study. The mean grade point average (GPA) with and without formative assessment was 2.83 (SD±.624745) and 2.29 (SD±.76575) respectively. There was a significant improvement in the overall GPA after the introduction of the formative assessments t (58) = 0.000567, p < .001.

Conclusion:

There was a significant improvement in the student’s academic performance after the implementation of formative assessments. Formative assessments consolidate the learning and reinforce the learning behavior. It is necessary that formative assessments should be incorporated into the regular assessment system for the optimal educational output.

## Background

Training the next generation of physicians is a tremendous undertaking that requires empirically supported learning methods and assessment techniques. Regular assessments in the medical education are intended to measure students’ knowledge, skills, and attitudes and, it is a requirement for a future practicing physician.

Two types of assessments used in the medical education are summative and formative assessments. Summative assessments evaluate a student’s learning progress and typically provide concrete grades or other objective measures ( Wallace, D. et al. 2016). The purpose of the summative assessments is to measure the students’ achievement to make decisions about promotion or progression, to direct what and how students learn, and to motivate students to learn ( Harlen and Deakin 2002). Formative assessments are used to reinforce the learning. It focuses on providing the student with feedback on their performance to improve their skills ( Cox, M. et al. 2007), knowledge, and learning behavior.

Summative assessments are comprehensive and usually conducted at the end of a course or a module. Formative assessments focus on specific content or topic or skills and can be conducted as frequently as possible. Formative assessments also serve the purpose to measure the student’s progress over time, which can help them to continuously improve and to motivate the students to learn. The objectives of formative assessments are to inform the students of required knowledge for the future, to identify student’s lack of performance, to assist with improvement in a timely fashion, and to provide feedback to teachers about how to assist the student. However, less is known regarding how formative assessment is related to other objective measures of student’s performance or less known about the impact of formative assessments on overall student’s performance in summative assessments.

## Introduction

This study was done at a Caribbean Medical School, Avalon University School of Medicine (AUSOM), Curacao which is offering four-year Doctor of Medicine (MD) program for bachelor’s graduates and 5-year premed/MD program for high school students. It has around 150 students on the basic sciences campus in Curacao and around 250 students in the clinical rotations are rotating at various teaching hospitals across the USA. Based on self-evaluation conducted in 2015 and 2016 at AUSOM, it was decided to move from a discipline-based curriculum to the integrated curriculum. The overall assessment strategy is revised at the time of change of the curriculum.

The dean of the medical school with his continuous quality improvement committee devised the robust, comprehensive, and authentic assessment strategy for the four-year MD program. The assessment plan is in line with the institutional learning objectives, teaching methods, and the content of the curriculum. Then individual course/clerkship chairs developed the assessment plan for each course and clinical rotations. Each course/clerkship assessment methods are based on the course content, teaching methods, and individual course/ clerkship learning objectives. Individual course/clerkship objectives are in turn based on the intuitional learning objectives to fulfill the mission and vision of the medical school. The alignment of learning objectives, instructional methods, and assessment methods is constructive alignment in the educational field ( Biggs, J. 1999). The assessment plan is presented to the curriculum committee for the final approval.

When developing assessment methods we took the importance of each area of knowledge, skills, and attitudes. Assessments formats were developed based on the understanding of Miller’s learning pyramid ( Miller, G. E. 1990). The “student knows” and “student knows how” can be tested by multiple choice questions (MCQs), oral examinations, and essay questions. “Student shows” can be tested by lab examinations, objective structured clinical exam (OSCE) using standardized patients, and clinical performance-based assessments. “Student does” can be assessed by patient encounters, and log books or work diaries. The assessments used to assess “student knows,” “student knows how,” and “student shows” are standardized assessments. “Student does” level assessments are unstandardized assessments.

Assessment methods are used which are valid and reliable. Validity and reliability are fully required for summative assessments. Although validity is a full requirement for formative assessments, reliability can be flexible (not a full requirement). All assessment methods that we use have educational effect; the assessments direct those who take it to prepare them in a way that is consistent with the educational goals of the course or program and catalytic effect; the assessments provide results and feedback in such a way that it creates and supports learning and acceptable for all stakeholders. To analyze the MCQs, we use MCQ’s item analysis by using P-value, bisserial (positive and negative bisserial) values, and horst static values. Blueprinting was developed for all courses. The blueprinting is developed in such a way that it is aligned with learning objectives of the course and are mapped by curricular mapping. Standardized examination procedures are in practice at Avalon. The compromised hofstee method is used for MCQ-based assessments and, borderline group method is used for performance-based assessments.

Formal formative assessments are introduced for the first time in AUSOM in January 2017 semester. In each basic sciences course, two formative assessments are mandatory in a semester. The faculty is allowed to conduct more than two formative assessments. In the clinical rotations, formative assessment and feedback are given in the form of mid-rotation assessment. General assessment methods that are used for formative assessments are MCQs, short answer questions, mind mapping/ concept mapping methods, flowcharts, oral examinations, objective structured practical examinations (OSPE), and standardized patient-based assessments. In clinical rotations, mid-rotation assessment or feedback is given based on the direct observation by the attending physicians and other healthcare professionals. The assessment methods that are being used for summative assessments are MCQs, oral examinations; objective structured practical examinations (OSPE), standardized patient-based assessments and objective structured clinical examinations (OSCE). The comprehensive clinical examination is given in the form of OSCE at the end of all core clinical rotations.

We had training sessions for our faculty members on assessment methods including formative assessments and summative assessments. Faculties were emphasized on giving effective and timely feedback. We trained the faculty members on how to give feedback by using Pendleton’s formula ( Pendleton, D. et al. 1984) after each formative assessment. In Pendleton’s formula of giving feedback, learner gets a chance to express his/her opinions regarding his/her strengths after the formative assessment. Then the faculty/instructor explains the strengths of the learner. Then learner can express his/her opinion regarding the weaknesses. Then the teacher explains the weaknesses of the learner. Then both learner and teacher will have a joint action plan to solve the weaknesses or combat the problems. Both of them having the partnership in solving the problems is crucial.

Avalon University developed a form for giving feedback to the students after each formative assessment. There are separate forms for giving feedback; one for basic sciences and another one for clinical rotations as mid-rotation assessment form. Faculty were instructed and informed on how to deliver the effective feedback. They were asked to be focused on that particular performance rather than generalized or vague feedback. Faculties were also informed to be centered around on how to improve the performance in the future and learning behaviors rather than criticism. A joint partnership of the instructor and the learner is given importance to own the plan of action which is aimed at improving the performance of the students.

The comments made by the faculty members will be reviewed by the Associate Dean of Basic Sciences, Clinical Dean, Curriculum committee and the Executive Dean. Faculty also made aware of the importance of PEARLS principles (Partnership for joint problem solving, Empathic understanding, Apology for barriers to the learner’s success, Respect for the learner’s values and choices, Legitimating of feelings and intentions of the learner, and Support for efforts at correction) while giving feedback during the faculty training workshop. Formative assessments foster the self-assessments and self-directed learning. Self-assessment and self-regulation of learning are important outcomes expected at higher education level.

The main problem associated with the formative assessments and giving effective and timely feedback in low resource setting is faculty’s time. Sometimes, there will be lack of sufficient numbers of faculty and, other time it is the availability of faculty’s time. So the faculty development activities play a crucial role in the successful implementation of formative assessments. We did conduct faculty development activities before implementing the formative assessments as explained above.

In our basic sciences program, our class size is around twenty-five to thirty students which enabled us to give the effective and timely feedback after each formative assessment. Each department in basic sciences has two or three faculty members. So we divide the class into groups and, each faculty member gets around ten students to give the feedback. In clinical rotations, every preceptor is allowed to have only two students. At any point of time faculty members in the clinical program are not allowed to have more than two students which made them give formative feedback in every rotation in the form of mid-rotation assessment in time and effective manner.

This study was done after one complete semester of implementation of formative assessments. We compared the student’s performance in summative assessments and their GPA in the semester with formative assessments and the previous semester where there are no formal formative assessments. This can be helpful in consolidating the fact that formative assessments facilitate and foster the learning process.

## Materials and Methods

A longitudinal cohort study was conducted among the basic sciences students at AUSOM in May 2017. The study was supported by quantitative data, and quantitative and qualitative questionnaires to address the objectives of the study. All the participants were provided with this set of questionnaires, graded on a 10-point scale. Students enrolled on basic sciences campus at the time of the study with more than 90 % of attendance were voluntarily included in the study.

Consent was obtained and, utmost priority was given to maintain the anonymity of the participants. AUSOM implemented and integrated formative assessment as an effective tool for evaluation into the curriculum from Jan 2017. Student grade point averages (GPA) were examined over two sequential semesters, without and with formative assessment. Students GPA were compared between September-December 2016 semester when there are no formal formative assessments with January-April 2017 semester when formative assessments are implemented. As formative evaluation was essentially used as an intervention, this study design was followed to test the effects of formative assessment on average grades in this population. Data were collected and analyzed using Stata 15 (Stata Corp.). The study proposal was presented and approved by the Reseach and Ethics Committee of Avalon University School of Medicine, Curacao.

## Results

58, among 67 total students took part in the study. The mean grade point average (GPA) with and without formative assessment was 2.83 (SD±.624745) and 2.29 (SD±.76575) respectively. 74 % (n=43) experienced an improvement in their GPA while 14 % (n=8) had lower GPA after the introduction and implementation of formative assessment and the rest 12% (n=7) being equivocal. There was a significant improvement in the overall GPA after the introduction of the formative assessment t (58) = 0.000567, p < .001.

### Students’ response

98.3% of the students were aware of the formative assessment, as an important tool for non-summative evaluation. 83.0% were convinced that formative assessment was helpful in achieving the learning objectives, and 81.0% believed that it strengthen their knowledge and understanding. Student’s responses and perspectives on the implementation and effectiveness of formative assessment were recorded and analyzed ( Table 1, 2). Majority of the responders (65.5%) responded that formative assessment was a strong predictor of self-evaluation.

**Table 1.** Students’ responses to the implementation of formative assessment (Based on a yes/no questions)

Students’ responses to the implementation of formative assessment	Yes	No
Are you aware of the formative assessment?	57(98.3%)	1(1.7%)
Is formative assessment helping in achieving the learning objectives?	48(83%)	10(17%)
Is formative assessment helpful in increasing your knowledge?	47(81%)	11(19%)
Does formative assessment motivate you in studying more?	44(71.6)	14(28.4%)
Are the feedback provided by the faculty members effective?	41(70.7%)	17(29.3%)
Are different types of questions used in formative assessments effective?	48(82.8%)	10(17.2%)
Is formative assessment a strong predictor of self- evaluation?	38(65.5%)	20(34.5%)

**Table 2.** Students perspectives on the implementation and effectiveness of formative assessment (Graded on a scale: 1-10)

	Mean (SD)	Median	Mode	Range
The effectiveness of formative assessment	6.9 (2.46)	7	8	2 - 10
Improvement after formative assessment	6.8 (2.44)	7	8	1 - 10
Provision of timely feedback	6.5 (2.80)	7	8	1 - 9
Satisfied with the faculty feedback	6.6 (2.82)	7	7	1 - 10

### Faculty feedback

62.7% students were satisfied with the faculty feedback after the formative assessment. The faculty feedback questions were consolidated with quantitative questionnaires on a scale of 10. The mean faculty satisfaction score was 6.6 (SD±2.82).

## Discussion

The role of assessment in medical education is not limited to medical school, but span’s a physician’s career as well ( Cilliers, F. et al. 2011). A central challenge in physician training is the lack of understanding regarding the effectiveness of formative assessments in medical training over time. Different types of assessment can be used to promote learning that reduces the likelihood of physician error in the future. Formative assessments can enhance the learning process if the feedback is provided effectively and timely manner. Research demonstrates that the combination of formative and summative assessments provides beneficial and long-lasting effects on learning in medical students ( Juwah, C. et al 2004; Veloski, J. et al 2006; Celebi, N. et al 2009; Holmboe, J. et al 2010) It is effective in consolidating the learning such that global gains are made and reflected in other objective measurements such as grade point average. This is a significant finding, which further supports the use of formative assessment within the context of medical education. Concrete knowledge of the association between formative assessment and grade point average in medical student education reinforces the effectiveness of its use and add to the existing literature that supports its efficacy.

It is imperative to strengthen and elucidate the role of formative assessment in medical education, particularly as it pertains to long-term competencies and expertise. If these assessment techniques are not studied thoroughly, they may not be efficacious in providing education to medical students, which will not be known without further study. While there have been significant advances in medical and pharmaceutical technology and research, this progress has not provided concrete answers regarding this question. A good balance of formative and summative assessments is required in the education and training to achieve a reasonable and comprehensive assessment strategy ( Juwah, C. et al 2004; Veloski, J. et al 2006; Celebi, N. et al 2009; Holmboe, J. et al 2010; Sharma, S. et al 2015).

## Conclusion

Proper assessment technique should be employed in the medical education to facilitate the learning process. There was a significant improvement in the student’s academic performance after the implementation of formative assessments. Formative assessments consolidate the learning and reinforce the learning behavior. It is necessary that formative assessments should be incorporated into the regular assessment strategy for the optimal educational output. Faculty development activities play a key role in providing the effective feedback and on time. The institutional policies and administration should emphasize on faculty development activities especially in the low resource setting where faculty numbers and time is a major constraint for the successful and effective implementation of formative assessments.

## Take Home Messages

• Faculty development activities are crucial for the successful implementation of formative assessments, especially in the low resource setting.
• Effective and timely feedback is the key to the formative assessments.
• Feedback needs to be focused on that particular performance rather than generalized or vague.
• Feedback needs to be centered around on how to improve the performance in the future and learning behaviors rather than criticism.
• Joint action plan by the instructor and the learner is essential for effective formative assessment implementation.

## Notes On Contributors

Dr. Sateesh Arja, MD, MPH, SFHEA, FAcadMEd, AFAMEE, Executive Dean and Director of Clinical Skills, Avalon University School of Medicine.

Dr. Yogesh Acharya, MD, FAcadMEd, Chair of Research and Ethics Committee, Avalon University School of Medicine.

Mr. Sabah Alezaireg, 3rd-year Medical Student,Avalon University School of Medicine.

Mr. Vishnupriyan Ilavarasan, 3rd-year Medical Student, Avalon University School of Medicine.

Mr. Samprith Ala, 3rd-year Medical Student, Avalon University School of Medicine.

Dr. Sireesha Bala Arja, MD, MPH, FHEA, FAcadMEd, Associate Professor of Pharmacology, Associate Dean of Basic Sciences.
