# Supplementary material for: Implementation of formative assessment and its effectiveness in undergraduate medical education: an experience at a Caribbean Medical School
Source: MedEdPublish (2016). 2018 Jun 13;7:131. [Version 1] doi: 10.15694/mep.2018.0000131.1 (PMC10699368; doi:10.15694/mep.2018.0000131.1)
Supplement: Peer review history

# Reviewer report: Reviewer response for version 1

**DOI:** 10.21956/mep.19601.r29111

**Published:** 2019-02-23

**Authors:** Trevor Gibbs

**Affiliations:** 1 AMEE

This review has been migrated. The reviewer awarded 3 stars out of 5 A well written paper about an important topic. One assumes that formative assessment is standard in most medical schools now and the value has been clearly demonstrated by other research. The way that formative assessment is structured is just as telling and for me, a clearer description of how it was carried out was missing from this paper- was it part of any portfolio, was it agreed and signed off etc.An interesting paper, well worth reading, but I think more relevant to the local setting in the hope that Avalon School can build on its findings.

Reviewer Expertise:

NA

No decision status is available

# Reviewer report: Reviewer response for version 1

**DOI:** 10.21956/mep.19601.r29110

**Published:** 2018-06-22

**Authors:** hussain shaik

**Affiliations:** 1 jjm medical college, india

This review has been migrated. The reviewer awarded 5 stars out of 5 It is very nice study, the method which tried in study is so effective, it make student stress free and so understandable, like this studies more should come to help Carrabian medical school students as well as faculty. thanks to the authors.

Reviewer Expertise:

NA

No decision status is available

# Reviewer report: Reviewer response for version 1

**DOI:** 10.21956/mep.19601.r29112

**Published:** 2018-06-16

**Authors:** P Ravi Shankar

**Affiliations:** 1 American International Medical University

This review has been migrated. The reviewer awarded 4 stars out of 5 I enjoyed reading this article on formative assessment from an offshore Caribbean medical school (OCMS). I am very happy that educational research is being conducted and published from OCMS. Formative assessments are being used to strengthen student learning at the Avalon University School of Medicine. The study is well described and easy to understand. I would be interested in knowing what methods are used for formative assessment and how feedback is provided to the students. The authors mention about a form which is used to provide feedback and I will be interested in knowing more details about the form. The authors could share in the Appendix the questionnaire used in the study. The present study is among basic science students and I hope the authors will publish about the impact of formative assessment among clinical students soon. The article is generally well-written, though, the language could be strengthened in a few places. I would like to congratulate the authors on their initiatives. The study will be of particular interest to educators in other resource constrained settings.

Reviewer Expertise:

NA

No decision status is available

# Reviewer report: Reviewer response for version 1

**DOI:** 10.21956/mep.19601.r29113

**Published:** 2018-06-15

**Authors:** SIVA PRASAD PALEM

**Affiliations:** 1 Basaveshwara Medical College and Hospital

This review has been migrated. The reviewer awarded 5 stars out of 5 This article is strongly recommended for medical faculty.

Reviewer Expertise:

NA

No decision status is available
